# Expertise paradigms for investigating the neural substrates of stable memories

**DOI:** 10.3389/fnhum.2013.00740

**Published:** 2013-11-01

**Authors:** Guillermo Campitelli, Craig Speelman

**Affiliations:** School of Psychology and Social Science, Edith Cowan UniversityPerth, WA, Australia

**Keywords:** stable memories, long-term memory, expertise, experimental paradigm, brain imaging

One of the hallmarks of acquiring expertise in any area of life is the ability to maintain relevant information over a long period of time (i.e., years or decades). Understanding the neural implementations of this ability requires the elucidation of two issues. First, the processes whereby recently acquired pieces of information become stable over time (i.e., memory consolidation); and second, the localization of these stable memories in the brain.

Unlike neurobiological and neuropsychological memory research, brain imaging research has paid little attention to these issues. Instead, most memory research in brain imaging has focused on the processes of memory encoding and retrieval. In this article we first succinctly present the current debate on the localization of stable memories in neurobiology and neuropsychology. We then discuss the difficulties in studying the localization of stable memories in human neuroimaging. After presenting the most traditional paradigm in studying long-term memory and the autobiographical memory paradigm, we present three expertise brain imaging paradigms. We also discuss how the latter help overcome the technical difficulties to investigate the neural localization of stable memories.

## Neurobiology and neuropsychology of stable memories

Memory consolidation has been intensively studied in mainstream neurobiology of memory with animal models (e.g., McGaugh, 2000; Dudai, 2004; Izquierdo et al., 2006). Moreover, neuropsychological studies of patients with brain lesions have been relevant to investigate the brain localization of stable memories (e.g., Milner et al., 1968; Squire and Alvarez, 1995; Nadel and Moscovitch, 1997).

A synthesis between these lines of research led to the development of two main models of the neural implementations of stable memories: the standard consolidation theory (SCT, Burnham, 1904; Squire and Alvarez, 1995; McGaugh, 2000; Dudai, 2004) and the multiple trace theory (MTT, Nadel and Moscovitch, 1997). A comparison between these theories is beyond the scope of this article (see Winocur et al., 2010, for further details). Suffice it to say that they agree on that, after acquiring new information, there is a period of *synaptic consolidation* that lasts from seconds to days, and that the hippocampus is essential in this process. They also agree on that there is a second type of consolidation—*system consolidation*—that lasts from months to decades, and that the result of this process is the formation of stable memories. However, there is no agreement whether these stable memories are localized in the hippocampus, in associative areas of the cortex, or in both.

## Difficulties in studying the localization of stable memories in brain imaging

Brain imaging studies are paramount to the testing of those theories in healthy humans. In their influential review on brain imaging, Cabeza and Nyberg (2000, p. 22) clearly indicated why this is a challenging endeavor:

“Encoding refers to processes that lead to the formation of new memory traces. Storage designates the maintenance of memory traces over time, including consolidation operations that make memory traces more permanent. Retrieval refers to the process of accessing stored memory traces. Encoding and retrieval processes are amenable to functional neuroimaging research, because they occur at specific points in time, whereas storage/consolidation processes are not, because they are temporally distributed (Buckner and Koutstaal, 1998).”

## Traditional paradigm

Long-term memory tasks typically involve a learning phase and a test phase. In the former, participants are presented with a set of items, and they are requested to memorize them. In the test phase participants are presented with items and they are requested to indicate whether or not each item was present in the previously presented set. The top row of Table 1 illustrates this approach. After subtracting the brain activation of a perceptual-motor control task, the activation due to the long-term memory task is believed to represent the neural correlates of long-term memory (e.g., Duncan et al., 2012).

**Table 1:** **Experimental paradigms to investigate neural correlates of long-term memory (LTM)**.

	**Main condition**		**Control condition**	
	**Task**	**Stimuli**	**Task**	**Stimuli**
Traditional paradigm	Learning-test	General	Perceptual-motor	General
Pre-scan interview	Test	Autobiographic	Test	General
Expert archival paradigm	Test	Domain specific (own)	Test	Domain specific (others)
Expert memory paradigm	Delayed response	Domain specific	Delayed response	General
Expert vs. novice paradigm	Simple domain-specific (experts)	Domain specific (experts)	Simple domain-specific (novices)	Domain specific (novices)

Brain imaging studies that use this traditional paradigm of long-term memory provide information on the neural implementations of how people learn new information, and how people retrieve information learned a few minutes or hours ago. However, the traditional paradigm fails to provide information on whether stable memories have a specific localization, and if so where such stable memories are localized in the brain.

## Autobiographical memory

The field of research that aims at filling this gap is the field of autobiographical memory. Instead of using a learning phase and a test phase, autobiographical memory studies use personal information provided by participants to generate experimental situations in which past information or experiences are retrieved during the experiment. For example, the pre-scan interview paradigm (e.g., Maguire and Mummery, 1999; see second row of Table 1) uses information gathered in a previous interview with participants to generate cues that would trigger past personal experiences. As a control condition participants answer general knowledge questions. One of the problems of this paradigm is that the brain activity during the experiment may reflect aspects of the interview, rather than the targeted past personal memories. A number of techniques have been proposed to overcome this problem, but they also have drawbacks. For example, Cabeza et al. (2004) proposed the “photo paradigm,” in which participants are given a camera to keep track of events of their lives. After a few days or weeks, participants are shown photos taken by them or other photos in a brain imaging session. The assumption is that the subtraction of the pattern of brain activation between these conditions would reveal the neural implementation of past personal memories. This paradigm solves the problem of contamination of memories from the interview, but it does not enable the study of remote memories.

Below we present three paradigms—the expert archival paradigm, the expert memory paradigm and the expert vs. novice paradigm—used in expertise studies that shed light on the neural localizations of stable memories. Memory theories based on expertise research (e.g., chunking theory, Chase and Simon, 1973; template theory, Gobet and Simon, 1996) emphasize the role of stable memories acquired through a period of practice of years or decades. Therefore, it is not surprising that brain imaging studies with experts have focused on the neural localization of these stable memories. We illustrate these paradigms with studies using chess players as participants.

## Expert archival paradigm

Given that experts learn domain-specific patterns, and that these patterns are stable memories, expertise studies aiming to uncover the brain localization of stable memories do not require a learning phase as in the traditional paradigm. Indeed, the learning phase occurred years ago. Moreover, if archival data is available, stimuli can be constructed, thus avoiding the interview of participants typical of autobiographical memory studies.

These features afford the possibility to design experiments with stimuli that would trigger the activation of well-consolidated memories. The third row of Table 1 illustrates this paradigm. For example, Campitelli et al. (2008) used the expert-archival paradigm, in which chess international masters were presented with positions of games they played in the past and positions belonging to other players. The task was to identify whether the positions belonged to their own games or not. In other words, this is a long-term memory task in which the learning phase occurred years before the experiment was conducted, and it is an autobiographical memory task in which a pre-scan interview was not necessary. The authors found a left-lateralized pattern of brain activation in the chess players. The pattern included activity at or near the left temporo-parietal junction, and a number of areas in the left frontal lobe, which is consistent with previous autobiographical memory studies (Maguire and Mummery, 1999; Maguire et al., 2000; Gilboa et al., 2004; Levine et al., 2004). The fact that the study with the expert archival paradigm showed similar results to the typical autobiographical memory paradigms provides evidence that the results found with the pre-scan interview are not an artifact of the paradigm.

## Expert memory paradigm

The previous paradigm sheds light on the neural localization of autobiographical stable memories. The expert memory paradigm helps understanding of the neural substrate of stable episodic and semantic memories. The expert memory paradigm also takes advantage of the fact that experts possess well consolidated memories of domain-specific patterns. It involves the comparison of experts' brain activity performing a task (e.g., a delayed-response task) using domain-specific stimuli and the same task with another type of stimuli. For example, Campitelli et al. (2007) compared the brain activity of chess experts performing a delayed-response task in two conditions: 1. stimuli were chess positions; and 2. stimuli were scenes with gray and white backgrounds and black and white shapes. This contrast is intriguing because it identifies the neural implementations of stable memories of domain-specific material by using a “working memory” task. This is because the working memory component of the delayed-response task is canceled out in the contrast. Incidentally, using the same task in this subtraction avoids the problem of “pure insertion” (i.e., the assumption that adding a process component to a task does not produce an interaction between the new component and other components of the task; Friston et al., 1996).

With this paradigm Campitelli et al. (2007) found activation in medial temporal areas. In a more localized study Bilalić et al. (2011b) found activity in a medial temporal area—the fusiform face area in the fusiform gyrus. This study provides evidence in favor of the view that this area is involved in expertise acquired to differentiate between members of the same class (e.g., Curby and Gauthier, 2010), as opposed to the view that this is an area specialized in processing faces (e.g., Kanwisher and Yovel, 2006).

## Expert vs. novice paradigm

The expert vs. novice paradigm, popularized by Chase and Simon (1973), can also shed light on the neural localization of stable memories. It involves recruiting a group of non-experts, who are requested to perform the same tasks as experts (i.e., a simple task of the domain of expertise). For example, Bilalić et al. (2011a) asked experts and novices to determine whether the king was in check in a chess position (see also Bilalić et al., 2010, 2011b, 2012, for similar approaches). A comparison of the brain activity in experts to that of non-experts affords the possibility of identifying whether stable memories are located in the same areas as not-so-well consolidated long-term memories. For example, Guida et al. (2012, 2013) conducted a review of expertise and training studies, and they identified that, in comparison to non-experts, when performing “working memory” tasks experts use less brain activity in working memory areas. Furthermore, experts show more activity than non-experts in long-term memory areas. These results support a two-stage model of neural implementations of expertise; the first stage involves efficiency in working-memory processing, and the second comprises a restructuring of brain areas involved in the consolidation of domain-specific long-term memories.

## Concluding remarks

We have described three expertise paradigms that have contributed to investigating the neural localization of stable memories. The expert archival paradigm aims at investigating the localization of stable autobiographical memories, the expert memory paradigm investigates the localization of episodic and semantic memories, and the expert vs. novice paradigm is important when investigating whether stable memories are localized in the same areas as the not-so-well consolidated long-term memories, or whether they become stable in other areas of the brain. An additional advantage of expertise paradigms is that they typically show large effect sizes, which increase the probability of finding statistically significant results.

Given that the ability to maintain relevant information over years or decades is apparent in domain-specific experts and everyday life experts these paradigms have the potential to shed light not only on the neural implementations of expertise, but also on the neural implementations of long-term memory in general.
